# Parliament and the Profession

**Published:** 1918-07-06

**Authors:** 


### Parliament and the Profession.

### Maternity and Child Welfare Bill.

This Bill, which extends the powers of local authorities in respect of maternity and child welfare work, was read a second time in the House of Commons on June 24.

### Dental Surgeons.

The Under-Secretary of State for War (Mr. Macpherson) informed Mr. Pennefather that, as far as can be ascertained, only two qualified dental surgeons are serving as privates, and they have not applied for commissions.

### Artificial Limbs.

Sir Arthur Griffith-Boscawen, Parliamentary Secretary to the Ministry of Pensions, informed Major Chapple that up to the end of May 18,307 men had been fitted, or were in process of being fitted, with artificial limbs, and 12,855 were still unfitted. Of the latter number, 4,321 were ready and waiting to be supplied.

### Doctors' Rations.

The Parliamentary Secretary to the Ministry of Food, Mr. Clynes, informed Mr. Arthur Richardson that civilian doctors in general practice receive the supplementary ration for heavy workers, in addition to the adult meat ration. In view of the quantities procurable under these combined rations no further increase is considered necessary.

### Mess Allowance of Nurses.

Major Chapple asked the Under-Secretary of State for War whether any advance in the mess allowance to nurses has recently been made to meet the increased cost of food; and whether he has satisfied himself that the increased and increasing strain being put upon nurses in the execution of their duties is being fully met by an adequate supply of nourishing food. Mr. Forster replied that an advance of 4s. 2d. was authorised in February 1917. He had no information to suggest that the nurses are suffering in consequence of an inadequate supply of nourishing food.

